# Human-Machine Interaction Methods for Minimally Invasive Surgical Robotic Arms

**DOI:** 10.1155/2022/9434725

**Published:** 2022-09-10

**Authors:** Fenglin Jiang, Ruiyao Jia, Xiujuan Jiang, Fang Cao, Tingting Lei, Li Luo

**Affiliations:** ^1^Chengdu Second People's Hospital, Chengdu 610000, China; ^2^Krirk University, Bangkok 10019, Thailand

## Abstract

Minimally invasive surgery has a smaller incision area than traditional open surgery, which can greatly reduce damage to the human body and improve the utilization of medical devices. However, minimally invasive surgery also has disadvantages such as limited flexibility and operational characteristics. The interactive minimally invasive surgical robot system not only improves the stability, safety, and accuracy of minimally invasive surgery but also introduces force feedback in controlling the surgical robot, which is a new development direction in the field of minimally invasive surgery. This paper reviews the development status of interactive minimally invasive surgical robotic systems and key technologies to achieve human-robot interaction and finally provides an outlook and summary of its development. Fuzzy theory and reinforcement learning are introduced into the parameter adjustment process of the variable guide control model, and a human-robot interaction method for minimally invasive surgical robot posture adjustment is proposed.

## 1. Introduction

Minimally invasive surgery has gradually become a hot spot for research in the field of surgery because of its advantages such as less intraoperative pain, smaller surgical incisions, lower chance of postoperative infection, and shorter recovery period. Robot-assisted minimally invasive surgery transforms the traditional bedside operation mode of minimally invasive surgery into a teleoperation mode based on the human-machine system, which also brings new challenges to surgeons. The uncoordinated hand-eye operation, narrow operation space, limited visual information (only the area within the endoscopic illumination range can be observed), lack of force feedback information, and imprecise movement of the robot arm caused by the unaided control will make the surgeon easy to overoperate during the operation, which will lead to collision and interference of the robot actuator and the end of the actuator out of the field of view. This not only increases the mental burden of the surgeon and affects the efficiency of the surgery but also poses the risk of causing secondary injuries to the patient, which brings safety problems that should not be underestimated [1–3]. Therefore, how to take measures to avoid such problems and improve the efficiency and safety of robot-assisted minimally invasive surgery is an important issue in the field of medical robotics research today.

In this paper, we introduce fuzzy theory and reinforcement learning into the parameter adjustment process of the variable guide control model and propose a human-robot interaction method for minimally invasive surgical robot posture adjustment and then build a guide parameter adjustment model containing individual operation characteristics in the joint space by online learning, in order to expect to obtain an adaptive human-robot interaction control strategy.

## 2. Description of the Problem

In robot-assisted minimally invasive surgery, the following three unexpected situations caused by human misoperation often occur due to the shortcomings of the robot master-slave teleoperation model.

### 2.1. Collision Interference between Patient Extracorporeal Robotic Arms

The main surgeon is located on the console side; only through monitoring, the image information of the patient's internal lesion is obtained but cannot grasp the movement of the patient's extracorporeal robot arm in real time; it is very easy for collision interference of the robot arm caused by excessive operation; the movement of the robot arm is blocked; the robot arm is stuck if not timely processing will cause damage to the motor [4, 5]. In this case, the operation is interrupted, and the assistant is needed to reposition the robot arm before continuing the operation, which affects the efficiency of the operation.

### 2.2. Collision Interference between the Surgical Instrument Rod and the Endoscope Rod in the Patient's Body Cavity

The endoscopic rod and the surgical instrument rod outside the endoscopic illumination area in the body cavity are not within the doctor's visual range, so the relative distance between them cannot be known during the operation. When the two collide and interfere, it may lead to damage to the surgical instruments or even inconsistent master-slave motion mapping due to the accidental movement of the endoscope head, which will lead to serious medical accidents.

### 2.3. The End of the Robot Actuator Is out of the Field of View

Due to the imprecision of the freehand operation and the uncertainty of the motion trajectory, the surgeon is likely to move the surgical tool outside the visual field during the operation. At this time, the medical staff cannot determine the exact position of the surgical tools in the patient's body, which may lead to damage to the organs and tissues outside the visualization range of the endoscope. Preoperative planning to find a reasonable incision position and initial robot arm position can greatly prevent this from happening in the effective operating space (within the area covering the lesion), but the robot actuator reach space is often larger than the actual demand, and it is impossible to completely prevent the actuator from moving outside the safe operating area, and the operating space for some procedures is narrow. Therefore, effective measures for human-robot interaction should be designed to ensure safe and smooth operation [6, 7].

## 3. Pendulum Control Algorithm

In this section, the specific implementation process of the main dynamic pendulum algorithm of the minimally invasive surgical robot is described in detail. The algorithm consists of two parts: the joint guide control model and the parameter adjustment model. The joint guide control model is used to establish the correspondence between the contact torque and the joint output velocity in order to realize the force interaction process between a human and a machine. The parameter adjustment model is mainly used for online learning and real-time adjustment of the guide model parameters, which are obtained by fuzzy Sarsa (*λ*) learning through online training.

### 3.1. Conductance Control Model

The impedance or admittance control model is the most common active compliance control method. Impedance control and conductance control complement each other; impedance control usually uses position as the model input to control the force or torque output, while conductance control uses force or torque as the input to control the position or velocity output of the robot arm [8, 9]. The one-dimensional admittance control model is defined as follows:

(1) $$\begin{array}{c} f_{h}=m\left(x-x_{\text{d}}\right)+c\left(x-x_{\text{d}}\right)+k\left(x-x_{\text{d}}\right), \end{array}$$

where *f*_*h*_ is the contact force applied to the robot arm, *x* is the end position of the robot arm in the Cartesian coordinate system, *m* is the virtual mass parameter, *c* is the virtual damping parameter, and *k* is the virtual stiffness parameter. Since the robot active joint pose requires the robot arm to move freely in its workspace without constraints, the corresponding desired position *x*_d_, velocity *x*_d_, acceleration *x*_d_, and virtual stiffness are set to 0, which leads to

(2) $$\begin{array}{c} f_{h}=mx+cx. \end{array}$$

Unlike the direct teaching approach of industrial robots, the active pose of minimally invasive surgical robots is designed to avoid intraoperative collisions between robotic arms and to ensure that minimally invasive instruments can obtain an effective working space and therefore tends to focus more on the independent attitude adjustment of each linkage of the robotic arm rather than the trajectory of the end-effector in Cartesian space. In order to facilitate the posture adjustment of the minimally invasive surgical robotic arm linkage, the pendulum movements of each active joint should be independent of each other and not affected by each other. In addition, the contact position between the operator and the robotic arm should not be constrained by a specific position [10]. It is clear that contact force detection by means of a six-dimensional force sensor at the end of the arm is not suitable for the active pose operation of minimally invasive surgical robots. For these reasons, and in order to address the new application environment, this paper integrates torque sensors at each drive joint of the robot arm to detect contact torque, allowing the operator to apply force to any position of the linkage in a more direct manner for posture adjustment. In the independent motion space of each active joint, equation (2) is modified accordingly, as shown in equation (3), to meet the practical needs of independent compliance control of each linkage.

(3) $$\begin{array}{c} \tau_{h}=m\theta +c\theta . \end{array}$$

where *τ*_*h*_ is the contact moment applied to the driving joint and *θ* is the joint position.

The selection of the control model parameters will determine the guide control characteristics, the virtual mass will affect the rate of change of speed and the stability of the system, while the human-machine interaction feeling is mainly determined by the virtual damping parameter. When the virtual damping parameter increases, the controllability of the operation increases and the required force is also increased. When the damping parameter is small, the robot arm can reach the target position quickly, and the operating experience is less laborious, but it is followed by poor controllability and operating accuracy.

Usually, it is necessary to reduce the virtual damping at the beginning acceleration stage of the motion to achieve a fast response of the control intention and increase the virtual damping at the end stage of the motion to improve the positioning accuracy of the action. Therefore, how to adjust the damping parameters reasonably according to the current state during the human-computer interaction and balance the two contradictions is the problem that needs to be solved by the variable conductance control strategy [11, 12].

### 3.2. Virtual Damping Parameter Adjustment Model

#### 3.2.1. Fuzzy Sarsa (*λ*) Learning Algorithm

Sarsa (*λ*) learning algorithm is a multistep time-difference-based strategy value iteration algorithm. If *S*={*s*_1_,…, *s*_*N*_} represents the set of environmental states and *A*={*a*_1_,…, *a*_*M*_} represents the output action set, then at any moment *t*, the intelligence selects and executes the action *a*_*t*_ ∈ *A*. According to the environmental state *s*_*t*_ ∈ *S* and the current strategy, the action *a*_*t*_ will have a certain impact on the environment at the next moment, and the environment then transforms to a new state *s*_*t*+1_ ∈ *S*, while the intelligence will receive the instantaneous return value *r*(*s*_*r*_+*a*_*r*_) ∈ *R* from the environment and update the action value function *Q*_*r*+1_(*s*_*r*_+*a*_*r*_). According to the return, the above steps are repeated in the learning process and the current policy is modified in an iterative manner to gradually approach the optimum.

Traditional reinforcement learning algorithms are generally applicable to discrete and finite state space descriptions and action outputs; however, many practical problems in reality have large or continuous state spaces, and in some cases, continuous action outputs can enhance the practical application of the algorithm. If reinforcement learning is applied to the pendulum adjustment process of a minimally invasive surgical robot, it is necessary to face the actual situation that the state space (velocity, acceleration, contact force, etc.) varies continuously and the action output (control model parameters) is required to be continuous [13–15]. The introduction of fuzzy theory into reinforcement learning can effectively solve these problems and can better respond to human intentions and help improve the interaction experience. Fuzzy Sarsa (*λ*) learning uses the concept of fuzzy sets to deal with continuous state input problems. The current environmental state is determined by both state variables *I*_*i*_(1 ≤ *i* ≤ *N*_*I*_) and fuzzy rules. The state quantity *I*_*i*_ is represented by *N*_*i*_ fuzzy sets in its theoretic domain *X*_*i*_, and the membership degree *μ*(*I*_*i*_) of the state variable *I*_*i*_ and the currently activated fuzzy state set *F*={*s*_1_,…, *s*_*n*_}, *n* < *N*, are obtained by the fuzzy state rules, where *N* is the spatial dimension divided by the fuzzy states. The degree of activation corresponding to each fuzzy state is calculated by the parametric number *T* (equation (5)), represented by the normalized weights *w*(*s*_*j*_), where the 1 ≤ *j* ≤ *n* fuzzified environmental state is used as the input for reinforcement learning *U*(*F*), and the correspondence between each fuzzy state division and the discrete action set A is established by continuous online training and the continuous action output *U*(*F*) for fuzzy Sarsa(*λ*) learning is calculated by weighted summation (equation (6)). It can be seen that the main role of fuzzy rules is to accomplish the recognition of continuous environmental state inputs at the input side of reinforcement learning and to achieve the linear integration of discrete actions for the output part of reinforcement learning [16, 17].

(4) $$\begin{array}{c} N=\prod_{i=1}^{N_{1}}N_{i}, \end{array}$$

(5) $$\begin{array}{c} w\left(s_{j}\right)=\frac{\prod_{i=1}^{N_{1}}\mu_{j}\left(I_{i}\right)}{\sum_{j=1}^{n}\prod_{i=1}^{N_{1}}\mu_{j}\left(I_{i}\right)}, \end{array}$$

(6) $$\begin{array}{c} U\left(F\right)=\sum_{j=1}^{n}a_{k}w\left(s_{j}\right),\;1\leq k\leq M. \end{array}$$

The action selection for Sarsa (*λ*) learning follows the same strategy as the update of the action value function. The discrete action selection in each fuzzy state is determined by the current exploration strategy according to the corresponding action value function, and the Boltzmann exploration strategy used in this paper is shown as follows:

(7) $$\begin{array}{c} P\left(a_{k}|s_{j}\right)=\frac{e^{Q_{t}\left(s_{j},a_{k}\right)}/T}{\sum_{k=1}^{M}e^{Q_{t}\left(s_{j},a_{k}\right)}/T}, \end{array}$$

where *P*(*a*_*k*_*|s*_*j*_) denotes the probability of selecting a discrete action *a*_*k*_ when the fuzzy state is *s*_*j*_. *T* is the temperature parameter, which is used to control the randomness of action selection. In order to reflect the long-term impact produced by the current action of the intelligence, the qualification trace function is used to realize part of the memory function of the intelligence to make the reinforcement learning more efficient as shown in the following formula:

(8) $$\begin{array}{c} e_{t}\left(s,a\right)=\left\{\begin{array}{cc} \gamma \lambda e_{t-1}\left(s,a\right)+w\left(s_{j}\right), & s=s_{j}\text{ and }a=a_{j}\\ \gamma \lambda e_{t-1}\left(s,a\right), & \text{otherwise} \end{array}\right\}, \end{array}$$

where *e*_*t*_(*s*, *a*) is the eligibility trace of the *t* momentary state-action pair, *γ* is the discount factor to weigh the impact of future returns on the current generation, and *λ* is the trajectory degradation parameter. After the execution action *a*_*j*_ corresponding to the fuzzy state *s*_*j*_ is selected, the eligibility traces of all state-action pairs are updated according to equation (8), i.e., the current state and the qualification traces of the action pair increase the corresponding weights and the rest decay proportionally [18]. Combining the eligibility traces, the action value functions of the state-action pairs are iteratively updated as follows:

(9) $$\begin{array}{c} Q_{\text{t}+1}\left(s_{j},a_{j}\right)=Q_{\text{t}}\left(s_{j},a_{j}\right)+\alpha \delta e_{t}\left(s_{t},a_{t}\right),\\ Q_{\text{t}}\left(F_{t},U_{t}\right)=\sum_{j=1}^{n}Q_{\text{t}}\left(s_{j},a_{j}\right)w\left(s_{j}\right),\\ \delta_{t}=r\left(F_{t},U_{t}\right)+\gamma Q_{\text{t}}\left(F_{t+1},U_{t+1}\right)-Q_{\text{t}}\left(F_{t},U_{t}\right), \end{array}$$

where *δ* is the time difference error, *r*(*F*, *U*) is the instantaneous return value, and *α* is the learning rate, which determines the proportion of the instantaneous return in the current *Q* value update.

#### 3.2.2. Return Function

In order to make the online training process of robotic arm pendulum operation not constrained by position, this paper only uses the robotic arm joint velocity, acceleration, and contact moment between a human and a machine as the state input variables for reinforcement learning and obtains the continuously changing environmental state during force interaction through state variables and fuzzy rules [19]. Since the virtual mass parameters in the conductance control model have much less influence on the operation feeling than the virtual damping, this paper sets the virtual mass parameters as constant values based on experience under the premise of ensuring the stability of the pendulum operation and takes the online adjustment of the virtual damping parameters as the main goal of fuzzy Sarsa (*λ*) learning, i.e., the action output set of fuzzy Sarsa (*λ*) learning is a number of discrete virtual damping values. The online learning process of the fuzzy Sarsa (*λ*) algorithm is actually to establish the optimal matching relationship between the fuzzy state inputs and the action outputs, and the so-called optimal matching relationship can be reflected by the payoff function. The goal of learning is to obtain the action execution policy that maximizes the cumulative payoff value of the whole learning process. Therefore, the payoff function can be defined according to the performance metrics that are expected to be optimized during the human-computer interaction [20]. The ideal human-machine force interaction approach is to expect the robotic arm to produce a soft and natural following motion as a human arm does for mobile operations. When a human-controlled arm performs a point-to-point movement task, it always instinctively minimizes the acceleration variation of the motion process, i.e., the cumulative value of the additive acceleration. In addition, the acceleration is also used as a smoothness indicator in the human-computer interaction of redundant robotic arms. In order to improve the operating perception during the active swing of the robotic arm and make the following motion of the robotic arm closer to the human operating characteristics, we want to optimize the above evaluation metrics by the reinforcement learning algorithm in an online learning manner. The instantaneous return function for fuzzy Sarsa (*λ*) learning is constructed as follows [21, 22]:

(10) $$\begin{array}{c} r\left(F,U\right)=-\sum_{k=t_{m}}^{t_{m+1}}\left|\overset{\ldots }{\theta_{k}}\right|, \end{array}$$

(11) $$\begin{array}{c} R=\sum_{t=0}^{t_{t}}r\left(F_{t},U_{t}\right), \end{array}$$

where *t*_*m*_ is the execution period of fuzzy Sarsa (*λ*) learning and $\left|\overset{\ldots }{\theta_{k}}\right|$ denotes the absolute value of joint plus acceleration. Online training by fuzzy Sarsa (*λ*) learning searches for the virtual damping parameter adjustment strategy that maximizes equation (11) based on the environmental state and instantaneous returns, i.e., minimizes the variation *R* of the accumulated acceleration throughout the operation, where *t*_*t*_ is the execution time of the pendulum operation.

## 4. Experiment and Analysis

### 4.1. Experimental Platform and Experimental Design

**Figure 1:**
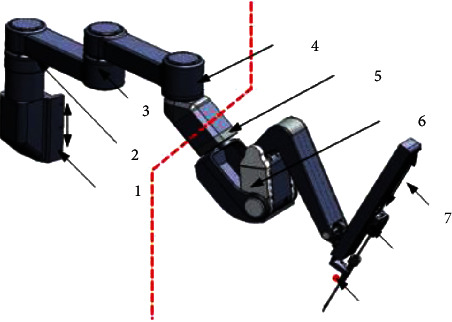
Structural design of the robotic arm of the surgical instruments.

In this section, the proposed variable conductance control algorithm will be verified by a self-developed minimally invasive surgical robotic arm.  Figure 1 shows the active control part of the arm, including two active rotating joints and one moving joint, using a real-time control system based on TwinCAT with an EtherCAT control cycle of 0.4 ms. Each active joint has an integrated torque sensor to detect the applied torque [23].

As shown in Figure 1, the posture adjustment of the minimally invasive surgical arm can be performed by dragging the two active joints in successive perpendicular directions of rotation. Since the contact force detection and control models of each joint are independent of each other, the relevant performance verification is carried out in this paper using drive joint 1 as an example [24]. The state variables of the joints (joint velocity *I*_1_, acceleration *I*_2_, and contact moment *I*_3_) are represented by five fuzzy sets in their respective theoretical domains, i.e., *N*_*i*_=5, *i*=1,2,3. The fuzzy sets are described by a triangular affiliation function, and their center-of-mass positions are uniformly and symmetrically distributed with 0 as the center. The adjustment range of the damping parameters can be roughly determined empirically by setting the corresponding discrete action set *A* = {0.11, 0.17, 0.23, 0.29, 0.35}. The algorithm execution cycle *t*_*m*_=4 ms, virtual mass parameter *m* = 0.25 kg, learning rate *α* = 0.01, discount factor *γ* = 0.95, and qualification trace degradation parameter *λ* = 0.95. The above reinforcement learning parameters are selected according to the actual test results.

In order to evaluate the actual performance of the active pendulum control algorithm proposed in the paper, three sets of comparison experiments are conducted, and the online training process of the corresponding virtual damping parameter tuning model is recorded, and when the algorithm converges to an approximately optimal strategy, it is compared and analyzed with the low damping value conductance model (*c* = 0.11), high damping value conductance model (*c* = 0.35), and variable damping model, respectively. In addition to the reinforcement to the performance metrics that need to be optimized for reinforcement learning, the operational accuracy and the energy required for the interaction process are also considered [25]. The positioning accuracy of the minimally invasive surgical manipulator arm can be obtained by measuring the maximum joint drift angle after the contact force disappears, and the energy required for the positioning process can be calculated by integrating the contact moment over the turning angle, i.e., ∫_0_^*t*_*t*_^|*τ*_*h*_|d*θ*.

### 4.2. Experimental Results and Analysis

**Figure 2:**
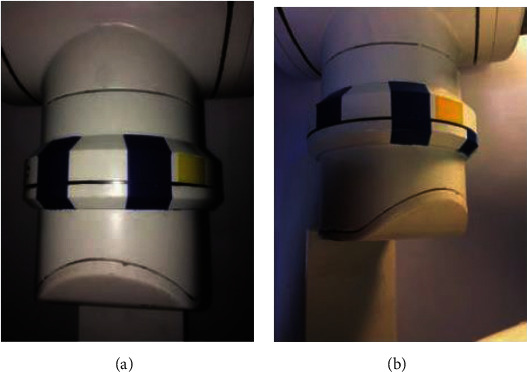
Online training position of the robotic arm. (a) Start position. (b) Stop position.

During the training process, participants turned the robotic arm from the starting position (double blue bar alignment position, −*π*/6) as shown in Figure 2(a) to the stopping position (single blue bar alignment position, *π*/6) as shown in Figure 2(b)according to their personal operating habits for a complete reinforcement learning training and repeated this process continuously until the instant of fuzzy Sarsa (*λ*) learning. The return value tends to be stable and the algorithm converges to an approximately optimal strategy, at which point the online training process of the variable derivative model ends.

In the online learning process of the variable conductance control model, with the increase of the training times, the changes of the virtual damping parameters gradually become clear from the chaotic state at the beginning, and the corresponding optimization indexes are also optimized. The return function converges to a fixed value after 21 independent training sessions (about 1 min), and the change process of the virtual damping parameters becomes stable. When the contact moment increases, the variable conductance control strategy automatically reduces the damping parameter value according to the current joint motion state, so that the motion speed of the robot arm changes faster and can quickly follow the motion trend of the arm in response to the human control intention, which makes the operation feel more effortless and easier to start. Conversely, when the contact force gradually decreases, the variable conductance control model increases the damping parameter accordingly to improve the positioning accuracy of the pendulum operation, assisting the operator to stop the robotic arm linkage at the desired posture position to reduce the overshoot and enhancing the safety of active compliance control, which is especially important for the pendulum operation of the minimally invasive surgical robotic arm [26]. At the same time, the fast convergence speed ensures the fast adaptation of the algorithm to different operator characteristics according to the experimental results of the minimally invasive surgical robotic arm pendulum control model comparison. For the same moving distance, the variable conductance control model based on fuzzy Sarsa (*λ*) learning is more energy-efficient than the high-damped-conductance model, with the maximum torque reduced from 2.72 Nm to 1.9 Nm, and the required energy decreased by 38.58%, while the positioning accuracy is very close to that of the high-damped-conductance model, with a significant improvement over the larger positioning overshoot of the low-damped-conductance model. Comparing with the variable guide parameter adjustment method, the damping parameter adjustment strategy optimized by the fuzzy Sarsa (*λ*) learning algorithm has a significant improvement in the control of acceleration fluctuations, which makes the active swing operation of the minimally invasive surgical arm more supple and natural [27–35].

## 5. Conclusion

In this paper, the active pose process of minimally invasive surgical robotic arm is implemented using variable conductance control. According to the actual requirements of robot-assisted minimally invasive surgery, a variable conductance control model oriented to the driving joints is designed, and each linkage of the minimally invasive surgical robotic arm can be adjusted independently for posture. Since the impact of virtual mass parameters on the human-robot interaction experience is minimal, this paper focuses on the study of adaptive variable damping methods. The human operating characteristics are taken into account in the online adjustment strategy of the virtual damping parameters through reinforcement learning and fuzzy theory. Combined with the experiments and the above analysis, it can be seen that the human-machine force interaction model proposed in this paper can respond well to the operator's control intention, effectively reduce the operation intensity, and has good flexibility, controllability, and rapid operator-oriented adaptation capability, which is suitable for the active positioning task of the minimally invasive surgical robotic arm. The adaptive adjustment strategy in this paper adopts the reinforcement learning method based on the fuzzy theory to train the guide parameters online, so the corresponding fuzzy space division and action set distribution will have some influence on the learning effect. In order to improve the online optimization efficiency of the algorithm and obtain a better human-computer interaction experience, the optimization of fuzzy set parameters will be the main research direction in the following.

## Data Availability

The dataset can be accessed upon request to the corresponding author.
